# Binary Ni-Co-Based Layered Double Hydroxide Nanoneedle Arrays for High Performance of Oxygen Evolution Reaction

**DOI:** 10.3390/nano13131941

**Published:** 2023-06-26

**Authors:** Zhi Lu, Zhihao Zhou, Shilin Li, Gongliang Tan, Hangtian Chen, Zishuo Ge, Chong Chen, Guangxin Wang

**Affiliations:** 1 School of Materials Science and Engineering, Henan University of Science and Technology, Luoyang 471003, China; a18338831577@163.com (Z.Z.); lishilin0402@163.com (S.L.); 15074792375@163.com (G.T.); hao17539591495@163.com (H.C.); 17838437881@163.com (Z.G.); chenchong02022@163.com (C.C.); 2 Henan Engineering Research Center for High Purity Materials and Sputtering Targets, Luoyang 471003, China; 3 Luoyang Key Laboratory of High Purity Materials and Sputtering Targets, Luoyang 471003, China

**Keywords:** nanoneedle, Ni-Co based, electrolysis, OER, electrocatalyst

## Abstract

Low-cost and high-performance electrocatalysts are crucial for water-splitting reactions. Some non-precious metal electrocatalysts are proved to be good replacements for noble metal due to the unique electronic structure features and excellent performance. In this work, binary Ni-Co-based layered double hydroxide nanoneedle arrays electrocatalysts are synthesized on Ni foam (NF) via a hydrothermal process. The microstructure and the catalytic performance of the catalyst changes significantly by regulating the molar ratio of Ni/Co. The theoretical analysis confirmed that the as-prepared NiCo-LDH nanoneedle arrays reveal a potential behavior in oxygen evolution reaction (OER) at a lower overpotential of 305 mV at 10.0 mA cm^−2^ and a Tafel slope of 110.38 mV dec^−1^. The double-layer capacitance (C_dl_) is 776 mF cm^−2^, which indicates that there are many active sites that are exposed on the surface for the electrocatalytic reaction. The results provide an obvious reference value to other types of LDH catalysts for the development of water electrolysis.

## 1. Introduction

To solve the problems of global warming and environmental pollution, the development and use of new energy is becoming more and more important. As the simplest form of energy carrier, hydrogen is considered to be the most excellent candidate to replace fossil fuels [1,2,3]. It has a high heat value and is convenient to transport and use, so it can be used in hydrogen internal combustion engines or in fuel cells [1]. Because the proportion of hydrogen in air is too little, the air separation is uneconomical [2]. Most of the hydrogen for industrial purposes is produced from the decomposition of the organic fuel, which brings huge energy consumption, enormous carbon emissions, and environmental pollution [3]. In this regard, the electrolysis of water is considered to be the most promising method for hydrogen production. The water-splitting reaction includes two half-reactions, which contain the hydrogen evolution reaction (HER) and oxygen evolution reaction (OER) [4,5]. As a sluggish kinetics process, OER is in a critical position in the reaction of water electrolysis [5,6,7]. Then, excellently designed OER catalysts become crucial in promoting the reaction efficiency of the electrolysis of water. For a long time, the catalysts were based on precious metals, for example, IrO_2_, Pt, and RuO_2_, which play a key role in raising the efficiency of HER and OER [8,9]. This is attributed to their unique electron shell structure [9], which can obviously lower the overpotential and accelerate the rate of electrolysis reaction. In practice, however, that means large costs to industrialized promotion [10,11]. Thus, it is urgent to research and synthesize excellent-performance OER electrocatalysts that are based on non-noble metals or low-cost nonmetallic materials [12,13].

Currently, abundant non-noble metal electrocatalysts have been synthesized, and significant progress has been made [14,15,16]. Among these non-noble metals, nickel foam has been widely used for the preparation of layered double hydroxide due to its high conductivity [17], corrosion resistance [18], relatively cheap [19], and especially its electronic orbit [20]. The oxides and hydroxide of many transition metals have been proved to have considerable activities to be used as catalysts [17]. The activities of these oxide and hydroxide are considered to have close relations with the number of 3d electrons of the transition metals atom; the d electron orbitals of surface transition metal ions show a bond to the anions on the metal surface [18,20]. This will affect the binding with the intermediates that contain oxygen; the bond strength of the intermediates is considered to have a decisive influence over the electrocatalytic activity. Understanding the correlation between the catalytic activities and the structure of the electronic orbit will contribute to the discovery of the mechanism of catalysts and the design of new cost-effective electrocatalysts for OER [20].

Ni-based LDHs have exhibited a good electrochemical property in electrocatalytic water splitting [17,18,19]. Firstly, this is attributed to the characteristic of the Ni atom orbit [20,21]. Jaramillo et al. found that the electronic structure of transition metal compounds leads to their excellent electrocatalytic performance [20]. Secondly, it is due to the corrosion resistance of nickel, which can remain stable upon prolonged exposure in oxidizing conditions [22]. Moreover, during the electrolysis of water, the surface ions of the metal can give a higher binding strength to the anion of the intermediates that offer the catalytic activity in the reaction [20]. 

Compared with most nanostructure catalysts, the large number of active sites and high specific surface area of LDH can be beneficial to the performance [23]. The high specific surface area may provide many charge-transfer paths to the reaction of water decomposition [24]. Nowadays, large kinds of LDHs are being designed, such as NiFe-LDH [25,26], Ni_x_Mg_3−x_Al-LDH/rGO [27], NiV-LDH [28], CoFe-LDH [29,30], and so on. Liu et al. designed a new strategy of high-entropy configuration to improve the activity and the stability of NiFe-LDH since these were decreased due to the Gibbs free energy and the Mg-O interfacial bond. These could keep the crystal structure more stable effectively and reduce the phase separation obviously; then, the LDH can give a dramatically stability with no activity decline at 300 mA cm^−2^ over 60 h [25]. Yu et al. designed Ni_x_Mg_3−x_Al-LDH/rGO catalysts with Au nanoclusters as precursors. The nanosheet array showed a crossover vertically on the side of rGO with Au nanoclusters dispersed on the edge. They found that the activity of the catalysts was connected to the Au clusters’ size and the ratio of Ni/Mg. The special nanostructure can improve the ion diffusion and transport during the reaction, which may give a new strategy for designing LDHs with a high performance for catalytic applications [27]. Chavan et al. prepared a NiV-LDH via the chemical bath deposition method. The electrocatalytic activity of this material was estimated both experimentally and theoretically. They found that the binding sites of the active O to the bands around the Fermi level improve the OER, leading to a good conductivity of the LDH. By doping V, the material structure could be regulated, and the ΔG of the O binding could be reduced. The hydrogen desorption energy of this LDH was low, and there was a large number of O catalytically active sites [28]. Badreldin et al. synthesized a series CoFe-based oxyhydroxides via a simple chemical method, and the ternary oxyhydroxides showed excellent performance and stability in alkaline, near-neutral, and neutral saline electrolytes [29].

Herein, we synthesized a NiCo-LDH nanoneedle on nickel foam via the hydrothermal method for the hydrolysis of water. This method is low-cost, and it is easy to regulate the microstructure even to the properties of the electrocatalyst. The special nanoneedle microstructure can give more active sites for the hydrolysis process. By controlling the ratio of Ni/Co, we adjusted the microstructure of the electrocatalyst. The correlation between the microstructure and the OER performance of the electrocatalyst was also investigated. The NiCo-LDH shows not only highly active but also long-term stability during the OER; a 305 mV overpotential under 10 mA cm^−2^ and a high C_dl_ of 776 mF cm^−2^ were obtained in 1 M KOH.

## 2. Materials and Methods

### 2.1. Materials

Co(NO_3_)_2_·6H_2_O, Ni(NO_3_)_2_·6H_2_O, and Ni foam (NF) were purchased from Aladdin. CO(NH_2_)_2_ and KOH were acquired from Sinopharm Group.

### 2.2. Electrocatalyst Synthesis

The Ni foams (1 cm × 2 cm) were treated by ultrasound in 1 M HCl for 20 min. They were then washed with ethyl alcohol and deionized water, in turn, three times to ensure that the surface oxides were removed. After that, the clean Ni foams were dried in a vacuum-drying chamber. Then NiCo-LDH nanoneedle arrays were synthesized on it via the hydrothermal method. A solution of Co(NO_3_)_2_·6H_2_O, CO(NH_2_)_2_, and Ni(NO_3_)_2_·6H_2_O was mixed into 60 mL ultrapure water under continuous stirring. The added molar ratio of the Ni:Co ion varied from 4:2 to 4:4 to 4:6 to 4:8. The urea was always 10 mmol. After stirring for 15 min, the pretreated Ni foam and the mixed liquids were moved to a 100 mL polytetrafluoroethylene reactor with steel casing and kept under 150 °C for 6 h. After the temperature dropped down to room temperature naturally, the obtained samples were washed with ethyl alcohol and ultrapure water, in turn, three times and were vacuum-dried under 60 °C overnight. 

After controlling the molar ratio of Ni:Co to optimize the OER performance of the electrocatalyst, the as-obtained specimens were marked as Ni_4_Co_2_-LDH, Ni_4_Co_4_-LDH, Ni_4_Co_6_-LDH, and Ni_4_Co_8_-LDH. 

### 2.3. Structural Characterization

An XRD diffractometer (D8 Advance, Bruker, Kyoto, Japan) with CuKα radiation was used to detect the phase of the as-obtained specimens. The scanning speed of the XRD diffractometer was 6°/min, and the 2θ range was 5–80 degrees. The morphology and EDS of the specimens were detected through a field emission scanning electron microscope, model JSM-7800F (JEOL Ltd., Tokyo, Japan), and a transmission electron microscopy, model TECNAI G2 F20 (FEI, New York, NY, USA). The specific surface areas of the samples were evaluated by a Brunauer–Emmett–Teller (BET) N_2_ adsorption–desorption solution model Micromeritics ASAP2460 (Norcross, GA, USA). The elemental composition and atomic states of the specimens were further examined by an X-ray photoelectron spectroscopy, model Thermo Scientific K-Alpha (Shanghai, China). 

### 2.4. Electrochemical Characterization

A three-electrode setup which was connected to a CHI660D electrochemical workstation (Tesco, Shanghai, China) was utilized for the electrochemical experiments under room temperature. A platinum electrode was used as a counter electrode, and an Ag/AgCl electrode was used as the reference electrode. The series of as-obtained electrocatalysts was used as the working electrode. The electrolyte was 1 M KOH. In this work, the working electrode potentials were converted to reversible hydrogen electrodes (RHEs) according to the following formula: E_RHE_ = E_Ag/AgCl_ + 0.1989 + 0.0591 × pH. The linear sweep voltammetry (LSV) curves were obtained under the scan rate of 2 mVs^−1^, and iR compensation was adopted for it. The calculation of overpotentials was performed according to the formula η(V) = E_RHE_ − 1.23. Electrochemical impedance spectroscopy (EIS) was tested under the sweep frequency between 0.01 and 10^4^ Hz. Cycle voltammetry (CV) was performed with a scanning speed ranging from 1.0 to 5.0 mV/s. Tafel slopes were tested under the same electrolyte. The electrochemical double-layer capacitance (C_dl_) of the specimens was tested through cycle voltammetry. A stability test was performed in virtue of chronoamperometry under 10 mA cm^−2^ and lasted for 24 h. 

## 3. Results and Discussion

### Structural Analysis

The Ni-Co LDH nanoneedle arrays have a considerable specific area, as shown in Table 1. The specific areas of Ni_4_Co_2_-LDH/NF, Ni_4_Co_4_-LDH/NF, Ni_4_Co_6_-LDH/NF, and Ni_4_Co_8_-LDH/NF are 8.6960 m^2^/g, 10.1848 m^2^/g, 11.9787 m^2^/g, and 7.3907 m^2^/g, respectively. The pore volumes of Ni_4_Co_2_-LDH/NF, Ni_4_Co_4_-LDH/NF, Ni_4_Co_6_-LDH/NF, and Ni_4_Co_8_-LDH/NF are 0.009751 cm^3^/g, 0.011258 cm^3^/g, 0.013901 cm^3^/g, and 0.008607 cm^3^/g. Thus, when the Ni/Co ratio is appropriately improved, the specific area and pore volume of NiCo-LDH/NF is increased obviously, thus helping to provide more active sites for OER and increase the catalytic efficiency.

**Figure 1:**
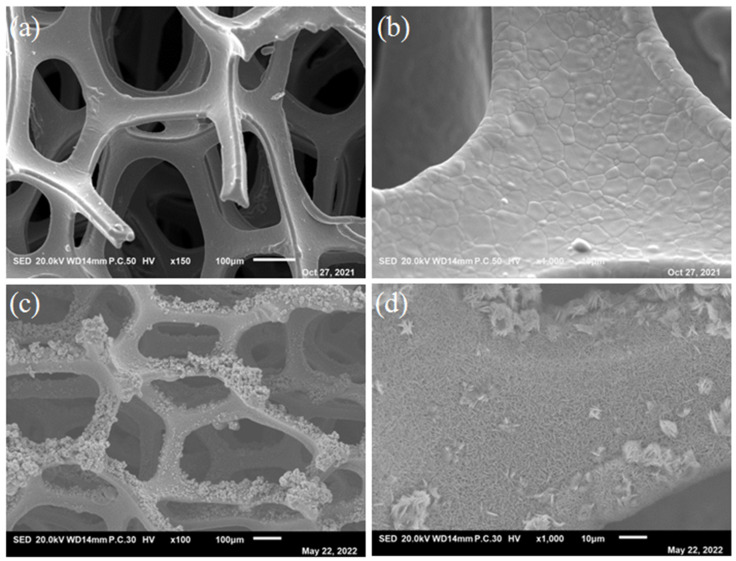
Low magnification of (**a**,**b**) NF before hydrothermal reaction and (**c**,**d**) Ni_4_Co_4_-LDH/NF after hydrothermal reaction.

The macro-morphology of the NF surface both before and after the synthesis is shown in Figure 1. It can be seen that, after the hydrothermal reaction, the Ni-Co LDH catalyst nanoneedle arrays grew uniformly on the three-dimensional network of NF substrate. The macroscopic porous structure of NF and the porous nanostructure of the specimens contributed to increasing the active surface area of the electrocatalyst significantly.

**Figure 2:**
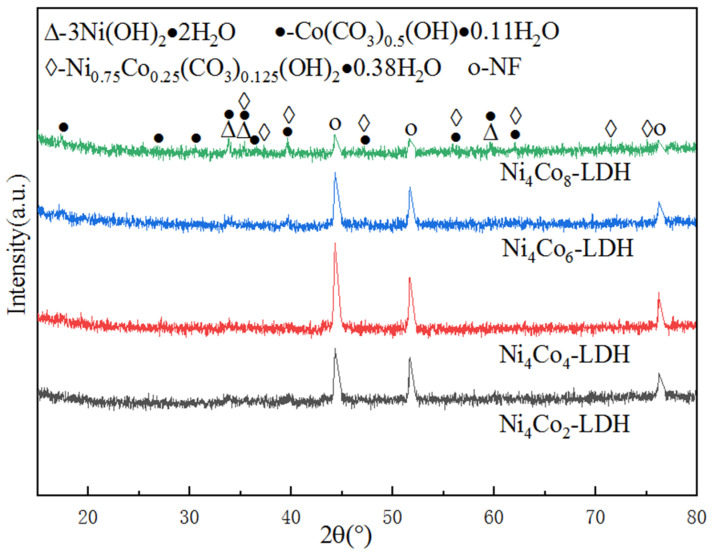
XRD patterns of as-prepared specimens.

Figure 2 shows the XRD patterns of the specimens. As shown in the patterns, there are three sharp peaks at 44.4°, 51.7°, and 76.3°; these correspond to NF(JCPDF No. 04-0850). The peaks located at 33.67, 35.19, and 59.60 belong to the (110), (111), and (300) lattice planes of 3Ni(OH)_2_·2H_2_O according to the PDF card JCPDF22-0444. Diffraction peaks at 34.95, 37.65, 39.40, and 62.44 represent the lattice planes (012), (104), (015), and (113) of Ni_0.75_Co_0.25_(CO_3_)_0.125_(OH)_2_·0.38H_2_O, according to the PDF card JCPDF40-0216.

The peaks at 17.51, 33.82, 35.48, 36.53, 39.53, 59.90, and 62.21 correspond to Co(CO_3_)_0.5_OH·0.11H_2_O of (020), (221), (040), (301), (231), (412), and (450), respectively, according to the PDF card JCPDF48-0083. As can be seen in Table 2, the FWHM value of Ni_4_Co_2_-LDH/NF is 0.306, and the crystallite size is 29 nm. Moreover, when the content of Co is increased, the FWHM value of Ni_4_Co_8_-LDH/NF reduces to 0.137, and the crystallite size increases to 89 nm, so the degree of crystallization is improved significantly. The binary layered double hydroxide was successfully synthesized onto the NF substrate.

**Figure 3:**
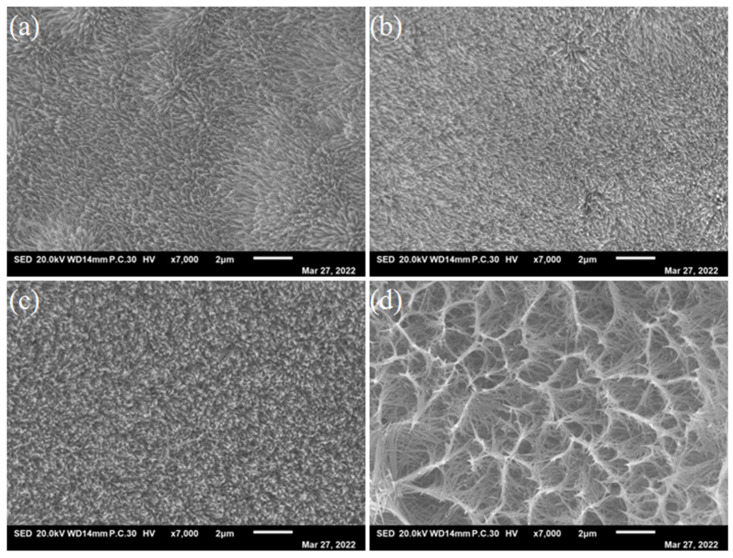
Low magnification of (**a**) Ni_4_Co_2_-LDH/NF, (**b**) Ni_4_Co_4_-LDH/NF, (**c**) Ni_4_Co_6_-LDH/NF, and (**d**) Ni_4_Co_8_-LDH/NF nanoneedle arrays.

**Figure 4:**
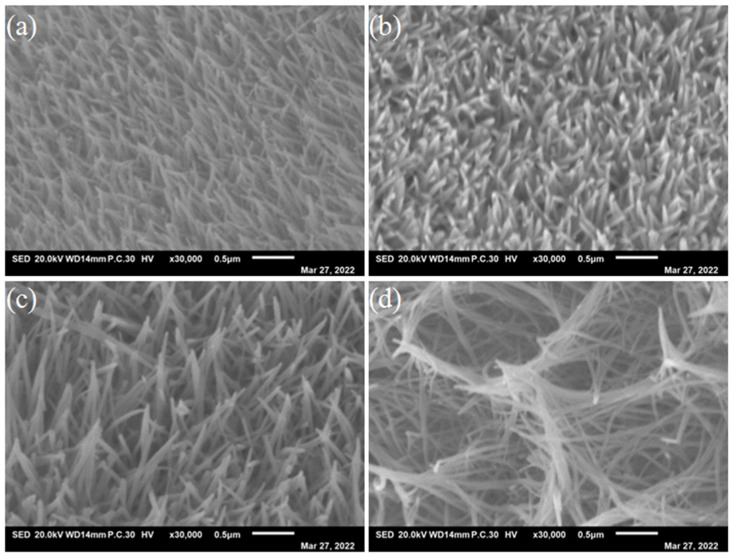
High magnification of (**a**) Ni_4_Co_2_-LDH/NF, (**b**) Ni_4_Co_4_-LDH/NF, (**c**) Ni_4_Co_6_-LDH/NF, and (**d**) Ni_4_Co_8_-LDH/NF nanoneedle arrays.

The macro-morphology of the specimens was observed by FESEM (Figure 3). As can be seen, the Ni-Co LDH presents nanoarrays. With a higher content of Co, the pores first decrease and then increase significantly. From the high-magnification morphologies of the samples in Figure 4, it can be seen that the as-prepared Ni-Co LDH electrocatalysts present nanoneedle arrays. Moreover, with the increasing amount of Co, the length of the nanoneedles increase obviously. Meanwhile, the porosity increases firstly and then decreases with the increasing Co, which corresponds to the pore volume information of Table 1. This may be in favor of improving the active surface area of the catalytic reaction, which contributes to the transfer of the charges between the catalysts and the electrolyte during OER [30] and is conducive to enhancing the properties of the electrocatalysts.

**Figure 5:**
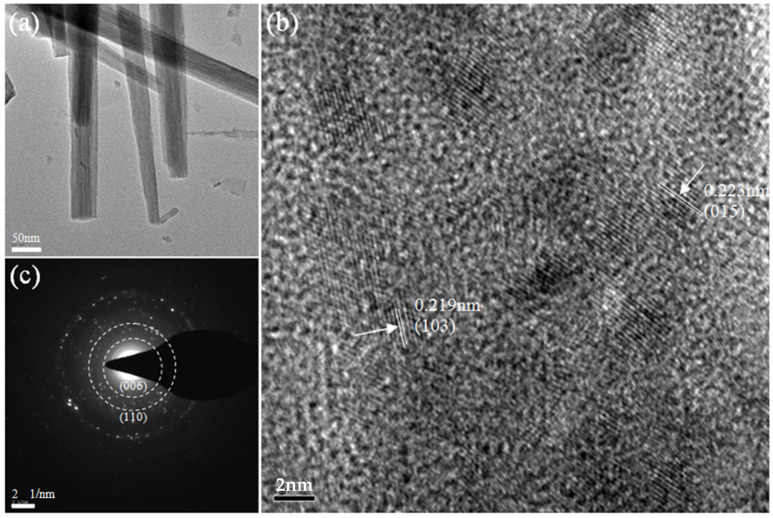
(**a**) Low-magnification TEM image, (**b**) high-resolution TEM image, and (**c**) selected-area electron diffraction of Ni_4_Co_4_-LDH/NF.

Figure 5 shows the TEM analysis of the Ni_4_Co_4_-LDH/NF. As can be seen in the figure, the nanoneedle morphology is clearly under the low-magnification TEM (Figure 5a). Figure 5b is the high-resolution picture of the microstructure of Ni_4_Co_4_-LDH/NF, in which the lattice fringes with distances of 0.219, and 0.223 nm correspond to the (103) crystal face of 3Ni(OH)_2_·2H_2_O and the (015) crystal plane of Ni_0.75_Co_0.25_(CO_3_)_0.125_(OH)_2_·0.38H_2_O. The corresponding SAED pattern (Figure 5c) shows the (110) crystal plane of 3Ni(OH)_2_·2H_2_O and the (006) crystal plane of Ni_0.75_Co_0.25_(CO_3_)_0.125_(OH)_2_·0.38H_2_O according to the JCPDF of X-ray diffraction (XRD).

**Figure 6:**
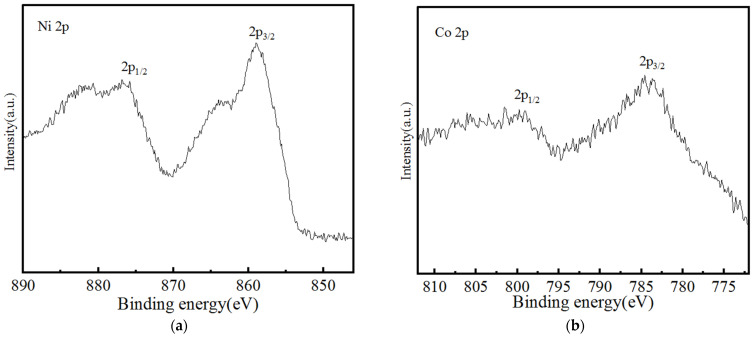
XPS spectra of Ni_4_Co_4_-LDH/NF: (**a**) Ni 2p and (**b**) Co 2p.

The chemical compositions and the atomic oxidation states of the material surface in the binary layered double hydroxide were investigated through X-ray photoelectron spectroscopy. Figure 6 is the XPS spectrum of Ni_4_Co_4_-LDH/NF, which reveals the distribution of the Ni and Co elements in the nanoneedle arrays’ surface. The peaks at 857.3 eV and 784.6 eV represent Ni 2p_3/2_ and Co 2p_3/2_ respectively, suggesting the +2 and +3 valence states of Ni and Co [31].

**Figure 7:**
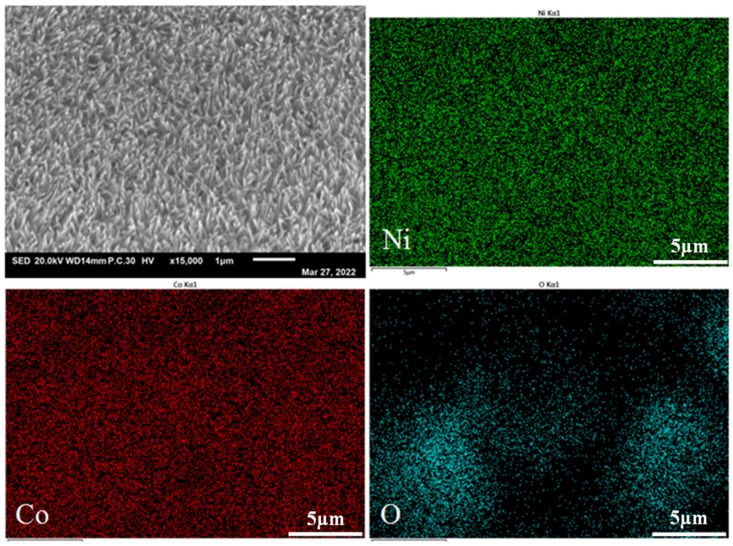

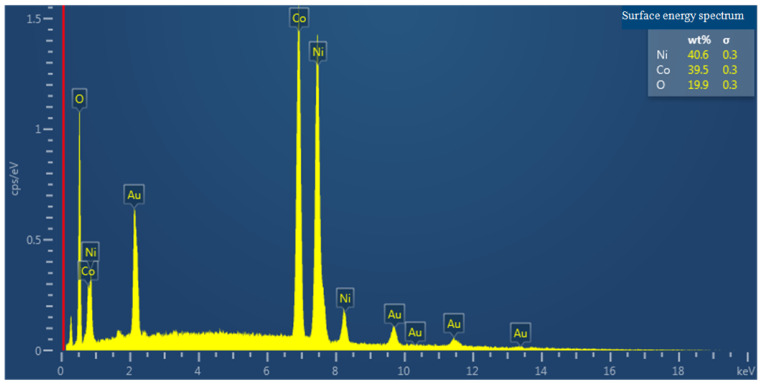
EDS of Ni_4_Co_4_-LDH/NF nanoneedle arrays.

Figure 7 shows the energy disperse spectroscopy of Ni_4_Co_4_-LDH/NF nanoneedle. As shown in the figure, the uniform distribution of Ni, Co, and O in the nanoarrays of the specimens confirms that the elements’ composition on the surface of Ni_4_Co_4_-LDH/NF is homogeneous; this contributes to the stability of the hydrolysis performance [32]. 

**Figure 8:**
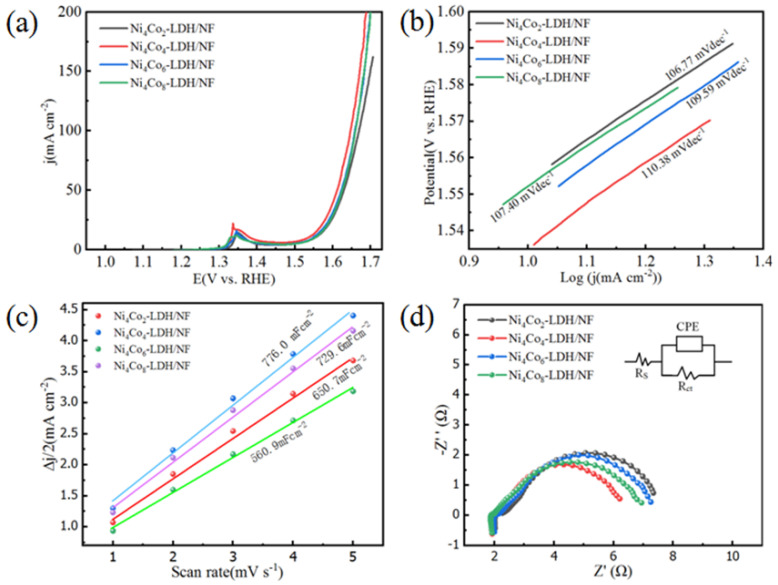
Polarization curves of NiCo-based LDH. (**a**) LSV plots of the specimens at 2 mVs^−1^ in 1 M KOH electrolyte. (**b**) Tafel slopes of the specimens. (**c**) Double-layer capacitances. (**d**) Electrochemical impedance spectroscopy Nyquist plots of the specimens. The inset diagram is the equivalent circuit.

The electrochemical characterization of the NiCo LDH series was investigated when it used as the working electrodes. The OER performances of the materials were studied in 1.0 M KOH. Figure 8 shows the electrochemical characterization of the NiCo LDH series electrocatalysts. Figure 8a displays the LSV polarization curve of the Ni-Co-based LDH. Ni_4_Co_4_-LDH/NF performs an overpotential of about 305 mV at 10 mAcm^−2^; this is lower than Ni_4_Co_2_-LDH/NF (324 mV), Ni_4_Co_6_-LDH/NF (318 mV), and Ni_4_Co_8_-LDH/NF (323 mV). To the interest of commercial water electrolysis, a higher current density is always required to keep for a considerable time to obtain commercial benefits. Therefore, it is important that a certified electrocatalyst can attain a demanded higher current density [29]. From the iR-corrected LSV polarization curve (Figure 8a), we can see that, to achieve the current density of 200 mAcm^−2^, the overpotentials of Ni_4_Co_2_-LDH/NF, Ni_4_Co_4_-LDH/NF, Ni_4_Co_6_-LDH/NF, and Ni_4_Co_8_-LDH/NF should be about 520 mV, 440 mV, 460 mV, and 467 mV. Thus, to achieve a higher current density, Ni_4_Co_4_-LDH/NF needs the lowest overpotential. Clearly, an appropriate Ni/Co elements ratio may decrease the overpotential significantly, which contributes to improving the catalytic behavior of the Ni-Co-based LDH [25,26]. 

Figure 7b shows the attained Tafel slopes tested in 1.0 M KOH. The series of NiCo-based LDHs shows relatively low Tafel slopes from 106.77 to 123.06 mVdec^−1^. Thus, the OER kinetics can be improved by the series of as-prepared NiCo-based LDHs. The double-layer capacitance (C_dl_) can be calculated from the CV scans [33]. Figure 8c shows the C_dl_ of the specimens. Ni_4_Co_4_-LDH/NF possesses a remarkably larger C_dl_ value (776 mF/cm^2^) than that of Ni_4_Co_2_-LDH/NF (650 mF/cm^2^), Ni_4_Co_6_-LDH/NF (561 mF/cm^2^), and Ni_4_Co_8_-LDH/NF (730 mF/cm^2^). This is attributable to the intrinsic activity and the exposure of more active reaction sites in the nanoneedle arrays of Ni_4_Co_4_-LDH/NF, which can enhance the OER behavior of the materials significantly [33,34].

EIS is tested to study the interfacial properties of the electrocatalysts electrodes [35]. It can be seen in Figure 8d that the equivalent circuit is simulated according to the Nyquist plots, in which R_ct_ represents the charge transfer resistance of the electrolytic system, R_s_ represents the solution resistance of the electrolytic system, and CPE is the constant phase angle elements of the electrolytic system. According to the EIS results, the R_ct_ of Ni_4_Co_4_-LDH/NF (1.983 Ω) is lower than that of Ni_4_Co_2_-LDH/NF (2.16 Ω) and Ni_4_Co_6_-LDH/NF (2.09 Ω) and nearly equal to that of Ni_4_Co_8_-LDH/NF (1.933 Ω). Thus, all the series of electrocatalysts display small resistances of 1.80–2.2 at 1.56 V vs. RHE, which may benefit from the high conductivity of the nickel foam skeleton [36]. This indicates the excellent electron transport kinetics of the series of NiCo LDHs [34,35,36].

As is well-known, a good OER performance (higher double-layered capacitance and lower charge transfer kinetics) is attributed to the defect that induced into the electronic structure and interface coordination associated with local charge distribution of the electrocatalysts. This was reached by doping Co. The Ni/Co ratio plays a critical role in determining the morphology of the Ni4Co4-LDH because the incorporation of Co atoms changes the growth kinetics [25,27,28]. Compared with Ni, Co has a relatively lower atomic weight in the precursor solution, so the reaction rate will be affected, and different surface morphologies will be obtained. It has been proved that electron transfer can be facilitated by the oxygen functional groups, which may hinder charge transfer between the electrolyte and electrode surface [28]. Therefore, when doping excess Co, the resistance to the charge transfer will be increased because there are increased oxidation states in the Co in the materials. Thus, the abundant oxidation states caused by doping excess Co and the increased number of active sites have a common influence on the OER activity of NiCo-LDHs [28]. 

**Figure 9:**
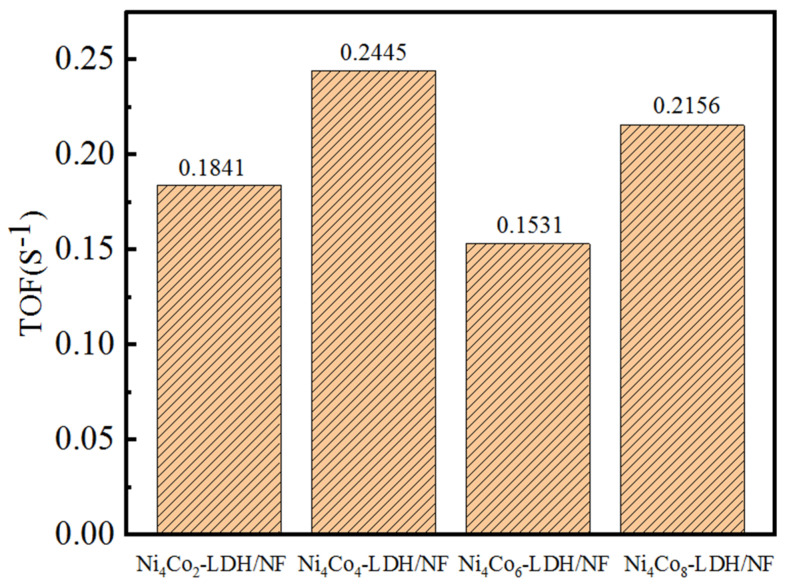
TOF values of the samples under the overpotential of 300 mV.

The catalytic activity of electrocatalysts is always evaluated through the turnover frequency (TOF). The calculation of the TOF is always performed according to the following formula: TOF = (AJ)/(4 mF), in which A represents the active area of the electrode materials, J represents the current density under 300 mV of overpotential, m represents the active sites number that estimated from the CV curves, and F is the Faradic constant [37,38]. According to Figure 9, the Ni_4_Co_4_-LDH/NF electrocatalyst presents a TOF value of 0.2445 S^−1^; this obviously exceeds that of Ni_4_Co_2_-LDH/NF (0.1841 S^−1^), Ni_4_Co_6_-LDH/NF (0.1531 S^−1^), and Ni_4_Co_8_-LDH/NF (0.2156 S^−1^). Therefore, Ni_4_Co_4_-LDH/NF has the best intrinsic catalytic activity when compared with the others, and this also means that the appreciable charge transfer efficiency contributed to the excellent performance of the OER [39].

The OER of the electrocatalyst is supposed to be composed of multistep reactions. Firstly, water molecules are adsorbed to the active sites on the catalysts, where the water molecules convert to some [OH] intermediate. After that, the intermediates will be oxidized and transformed to [O]. In the next step, [OOH] intermediate will be produced by the reaction between the [O] and H_2_O molecule. Finally, [OOH] will release O_2_. These intermediates have abundant oxygen vacancy sites on the electrocatalysts. Thus, the performance of OER is closely related to the amount of the active catalytic sites and the adsorption capacity between the intermediates and H_2_O [40].

**Figure 10:**
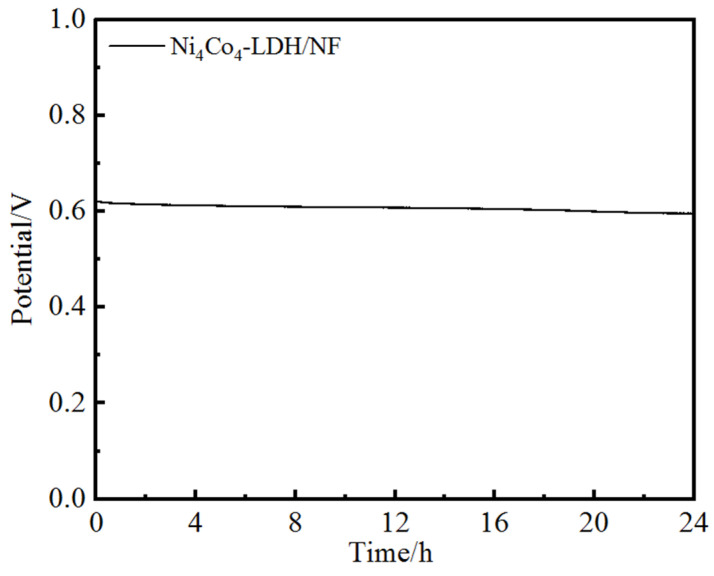
Chronoamperometry test of Ni_4_Co_4_-LDH/NF at 10 mA·cm^−2^.

For the application of the electrocatalysts, the long-time stability is a critical factor. The chronoamperometry is used to evaluate the long-time stability of the as-prepared electrocatalysts. The stability of Ni_4_Co_4_-LDH/NF was tested at a constant 10 mAcm^−2^ in the 1 M KOH. Figure 10 is the V-T curve of the 24 h stability test of Ni_4_Co_4_-LDH/NF. The potential of Ni_4_Co_4_-LDH/NF is stable and is kept for 24 h just with only a minor decrease from 0.62 to 0.59 V, suggesting a negligible activity loss of the catalyst after 24 h of testing [32]. This indicates that Ni_4_Co_4_-LDH/NF has excellent OER stability as an electrocatalyst.

The OER performances of the electrocatalysts synthesized in this paper and other reported Ni-based or Co-based LDH, electrocatalysts which show obvious advantages, are shown in Table 3. According to the references in the table, the reported Ni-based or Co-based LDH electrocatalysts are normally synthesized through a multi-step process and are costly in energy and time [41,42,43,44,45,46]. Meanwhile, in this work, the Ni-Co-based LDH electrocatalysts can be obtained by the one-step hydrothermal method, which is low in cost and easier to operate. Moreover, the overpotential at 10 mAcm^−2^ in this paper is superior to the works listed in the table, and this may be attribute to the nanoneedle arrays of the Ni-Co-based LDH. The abundant active sites can significantly promote the electron transport rate between the electrocatalysts and electrolyte, which contribute to the excellent OER behavior [44,45,46].

## 4. Conclusions

NiCo LDH nanoneedle arrays were synthesized on Ni foam through the hydrothermal method. The obtained NiCo-LDH nanoneedle arrays displayed long-time stability, in addition to an excellent catalytic property for OER. A lower overpotential of 305 mV and a Tafel slope of 110.38 mVdec^−1^ were obtained when Ni_4_Co_4_-LDH/NF was used in the electrolysis of water at 10 mAcm^−2^.

A remarkably larger C_dl_ value of 776 mF/cm^2^ and TOF values of 0.2445 S^−1^ were also obtained. This is better than most of the other Ni-based or Co-based LDH electrocatalysts that have been reported. The good behavior of the as-prepared specimen is owed to the microstructure of the materials. We believe that the method in this work can be further extended to other types of LDH catalysts for application in fields of energy storage and conversion.

## Figures and Tables

**Table 1:** Specific area and pore volume of the samples (the superior and inferior are the errors).

Sample	S_BET_ (m^2^/g)	V_total_ (cm^3^/g)
Ni_4_Co_2_-LDH/NF	8.6960$\begin{array}{c} +0.0047\\ -0.0034 \end{array}$	0.009751$\begin{array}{c} +0.000131\\ -0.001074 \end{array}$
Ni_4_Co_4_-LDH/NF	10.1848$\begin{array}{c} +0.0152\\ -0.0179 \end{array}$	0.011258$\begin{array}{c} +0.001038\\ -0.000495 \end{array}$
Ni_4_Co_6_-LDH/NF	11.9787$\begin{array}{c} +0.0114\\ -0.0093 \end{array}$	0.013901$\begin{array}{c} +0.000350\\ -0.002471 \end{array}$
Ni_4_Co_8_-LDH/NF	7.3907$\begin{array}{c} +0.0083\\ -0.0146 \end{array}$	0.008607$\begin{array}{c} +0.001670\\ -0.000930 \end{array}$

**Table 2:** FWHM and crystallite size of the samples.

Sample	FWHM	Crystallite Size (nm)
Ni_4_Co_2_-LDH/NF	0.306	29
Ni_4_Co_4_-LDH/NF	0.220	43
Ni_4_Co_6_-LDH/NF	0.187	68
Ni_4_Co_8_-LDH/NF	0.137	89

**Table 3:** OER performances of other NiCo-LDH electrocatalytic materials reported.

Catalyst	Current Density (mA cm^−2^)	Overpotential (mV)	Reference
Ni(OH)_2_	10	595	[41]
NiCo hydroxide	10	460	[39]
Co(OH)_2_/NF	10	280	[42]
NiCo-LDH	10	367	[43]
NiCo-NS	10	334	[44]
Co(OH)_2_	10	360	[45]
ZIF-67/CoNiAl-LDH/NF	10	303	[46]
Ni-Co-based LDH arrays	10	305	This work

## Data Availability

Not applicable.
